# Changes in parents' psychotropic medication use following child's cancer diagnosis: A fixed‐effects register‐study in Finland

**DOI:** 10.1002/cam4.4662

**Published:** 2022-03-28

**Authors:** Niina S. Metsä‐Simola, Hanna M. Remes, Elina M. Hiltunen, Pekka T. Martikainen

**Affiliations:** ^1^ Population Research Unit University of Helsinki Helsinki Finland; ^2^ Department of Public Health Sciences Stockholm University Stockholm Sweden; ^3^ Laboratory of Public Health Max Planck Institute for Demographic Research Rostock Germany

**Keywords:** caregiver burden, caregivers, childhood cancer, mental health, parents, population register, psychotropic drugs

## Abstract

**Background:**

Symptoms of depression and anxiety are elevated among parents of children with cancer. However, knowledge of parents' psychotropic medication use following child's cancer diagnosis is scarce.

**Methods:**

We use longitudinal Finnish register data on 3266 mothers and 2687 fathers whose child (aged 0–19) was diagnosed with cancer during 2000–2016. We record mothers' and fathers' psychotropic medication use (at least one annual purchase of anxiolytics, hypnotics, sedatives, or antidepressants) 5 years before and after the child's diagnosis and assess within‐individual changes in medication use by time since diagnosis, cancer type, child's age, presence of siblings, and parent's living arrangements and education using linear probability models with the individual fixed‐effects estimator. The fixed‐effects models compare each parent's annual probability of psychotropic medication use after diagnosis to their annual probability of medication use during the 5‐year period before the diagnosis.

**Results:**

Psychotropic medication use was more common among mothers than fathers already before the child's diagnosis, 11.2% versus 7.3%. Immediately after diagnosis, psychotropic medication use increased by 6.0 (95% CI 4.8–7.2) percentage points among mothers and by 3.2 (CI 2.1–4.2) percentage points among fathers. Among fathers, medication use returned to pre‐diagnosis level by the second year, except among those whose child was diagnosed with acute lymphoblastic leukemia or lymphoblastic lymphoma. Among mothers of children with a central nervous system cancer, medication use remained persistently elevated during the 5‐year follow‐up. For mothers with other under‐aged children or whose diagnosed child was younger than 10 years, the return to pre‐diagnosis level was also slow.

**Conclusions:**

Having a child with cancer clearly increases parents' psychotropic medication use. The increase is smaller and more short‐lived among fathers, but among mothers its duration depends on both cancer type and family characteristics. Our results suggest that an increased care burden poses particular strain to the long‐term mental well‐being of mothers.

## Graphical abstract

Parents’ psychotropic medication use clearly increases after their child’s cancer diagnosis, the increase being larger among mothers.The increase is short‐lived among fathers, but among mothers its duration depends on both cancer type and family characteristics related to caregiving needs.

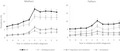

## 1 INTRODUCTION

Having a child with cancer increases psychological distress among parents.^1^, ^2^, ^3^ A recent meta‐analysis estimated a pooled prevalence of 21% for anxiety and 28% for moderate to high depression among parents of children previously diagnosed with cancer, but there was very high heterogeneity in the prevalence estimates between previous studies.^4^ Most longitudinal studies suggest that parents' symptoms generally decline from the high level observed at the time of their child's diagnosis during the following years, but a considerable proportion of parents report prolonged distress, the predictors of which are not well known.^2^, ^5^, ^6^, ^7^, ^8^, ^9^, ^10^, ^11^ The magnitude and duration of psychological distress are likely to vary depending on how parents cope with the immediate shock and longer‐term stress related to their child's diagnosis of severe illness, and the potential experience of caregiving strain arising from difficulties in combining increased care demands with other responsibilities.^3^, ^12^, ^13^, ^14^ Survival rates for childhood cancers have generally improved, but the risk of long‐term adverse effects (and also death) varies depending on the type of cancer,^15^, ^16^, ^17^, ^18^, ^19^ with central nervous system (CNS) tumors in particular characterized by both high treatment burden and effects on the CNS.^20^ Uncertainty is considered particularly stressful,^3^, ^12^, ^21^ and cancers with poor prognosis and high uncertainty of outcome may thus particularly increase the probability of parental distress. Complicated childhood cancers that require frequent hospital care, with parents spending prolonged periods of time in the hospital, have a particularly strong adverse effect on parental mental health,^10^, ^12^, ^21^, ^22^, ^23^ and caregiving strain may also remain elevated during off‐treatment periods.^24^

Apart from the type of cancer, the amount of caregiving strain that parents experience is likely to depend on the child's age at diagnosis and other family characteristics. A child's younger age at diagnosis has been found to increase parental distress,^25^ potentially because younger children need more care irrespective of their treatment status. The presence of other underage siblings is also likely to increase care demands on parents. Difficulties in balancing caregiving needs with other responsibilities may affect parents' employment and income adversely,^26^, ^27^ which can further increase psychological distress among parents of children with cancer.^21^, ^28^, ^29^ The opportunities to combine family and work are likely shaped by education level, as education is related to both job demands and flexibility, which are known to affect the balance between family and work among parents of ill children.^14^, ^30^, ^31^ Across Europe, women usually provide more childcare than men,^32^ and parents of children with cancer often follow traditional gender roles when dividing parental tasks.^33^ Mothers are thus more likely than fathers to experience problems with balancing family and work,^14^ and particularly during active treatment mothers of children with cancer have reported higher levels of distress compared to fathers.^2^ These problems may be most pronounced among single‐mothers, who are on average socioeconomically less advantaged,^34^ and also lack support from the other parent. Lack of social support is a known predictor of psychological symptoms,^35^ and single‐parents with low levels of support may be particularly vulnerable to adverse mental health effects when their child is diagnosed with cancer.^36^ Nevertheless, a comprehensive understanding of parental and family characteristics that predict prolonged psychological distress among parents of children diagnosed with cancer is still lacking.

Most previous studies on parents' mental well‐being after child's cancer diagnosis have focused on self‐reported symptoms of psychological distress.^2^, ^3^, ^8^ While self‐reports describe the parents' subjective experience of psychological distress, population‐level studies with clinical outcomes provide reliable information on the prevalence of symptoms that are severe enough to warrant medical treatment. The few previous studies using clinical measures have focused exclusively on the first occurrence of using mental healthcare services, either on first psychotropic medication purchase,^37^ first hospital contact for any psychiatric disorder,^25^ or first contact with mental healthcare.^38^ A Danish register‐study showed that during the first year since child's cancer diagnosis parents' risk of starting psychotropic medication use was clearly elevated, but the study only examined the risk of first prescription, and did not report on changes in psychotropic medication use over time.^37^ In two other recent register‐based studies, mothers' probability of receiving mental healthcare remained elevated for at least 20 years after the child's initial diagnosis, but neither study specifically examined changes in the prevalence of mental health symptoms according to time since the child's diagnosis, nor the predictors of prolonged use of mental healthcare.^25^, ^38^

To examine both the onset and duration of parents' psychological distress using a clinical measure of mental health, we take advantage of population‐representative longitudinal register data and a fixed‐effects study design. We use information on parents' psychotropic medication use––measured by medication purchases––both before and after the child's cancer diagnosis to (1) estimate changes in mothers' and fathers' psychotropic medication use over time, and (2) assess whether these associations differ by cancer type, child's age at diagnosis, presence of an underage sibling, and parent's living arrangement and education. The fixed‐effects approach allows us to control our analyses not only for observed differences, but also for stable unobserved heterogeneity––such as parent's previous history of mental health problems and psychotropic medication use––between individuals.^39^

## 2 MATERIALS AND METHODS

We used longitudinal Finnish register data on all parents whose biological child was diagnosed with cancer during 2000–2016 at the age of 0–19 years. Information on cancers was derived from the Finnish Cancer Registry. Statistics Finland provided annual information on the sociodemographic and family characteristics of the children and their parents, and the Social Insurance Institution provided information on all psychotropic medication purchases. The data linkage was performed by Statistics Finland using personal identification codes assigned to all permanent residents, and researchers were allowed access to pseudonymized data (the Ethics Committee of Statistics Finland's permission TK‐53‐1121‐18).

From the Cancer Registry, we identified 3948 individuals aged 0–19 years at their first cancer diagnosis. Of them, 3935 could be linked to their biological mother and 3863 to their biological father. After excluding parents with more than one child diagnosed with cancer, our sample comprised 3892 mothers and 3822 fathers (see Figure S1 for all exclusions). We excluded 26 mothers and 51 fathers who were not living in Finland for at least 1 year before and 1 year after their child's diagnosis, and 33 mothers and 65 fathers who were deceased by the year of their child's diagnosis. Furthermore, 236 mothers and 227 fathers who lost their child within a year of diagnosis were excluded, because the mental health effect of having a child with cancer could not be distinguished from bereavement. A previous study using Finnish registry data has shown that parents' psychotropic medication use increases following the death of their child, with highest prevalence observed around 1 year after bereavement and followed by a steady decrease thereafter.^40^

The remaining 3597 mothers and 3479 fathers were followed for purchases of anxiolytics (ATC codes N05B), hypnotics and sedatives (N05C), and antidepressants (N06A)––herein referred to as psychotropic medication use––5 years before and 5 years after the exact date of their child's first cancer diagnosis. Drugs in the selected ATC categories are commonly used to treat anxiety, depression, insomnia, and related mental health conditions.^41^, ^42^, ^43^ For each year of follow‐up, we identified whether at least one psychotropic medication purchase had been made. In Finland, all psychotropic medications are prescribed by clinical doctors, and all permanent residents are entitled to reimbursement for prescription medication expenses. The reimbursement is automatically deducted from the price of the medication at the pharmacy, and the purchase recorded in the prescription register maintained by the Social Insurance Institution. The prescription register includes detailed information on the date of purchase and type of purchased medication, but for simplicity, we used an annual‐level measure of any purchase of anxiolytics, hypnotics and sedatives, or antidepressants.

We censored parents whose child died (361 mothers, 350 fathers) or who themselves died (37 mothers, 84 fathers), or emigrated (13 mothers, 15 fathers) between the second and fifth year since the child's diagnosis at the year of death / emigration. Preliminary analyses showed that psychotropic medication use did not vary around the time of child's cancer among nonresident fathers, whereas nonresident mothers were too few for a reliable sub‐group analysis (Table S1). We thus excluded all nonresident parents (331 mothers, 792 fathers), leaving us with a final sample of 3266 mothers and 2687 fathers. The higher number of nonresident fathers compared to nonresident mothers results from most children residing with their mothers after parental separation.

Time since child's cancer diagnosis was divided into categories and coded as 0 during the 5 years before the date of diagnosis, and 1 during the first, 2 during the second and third, and 3 during the fourth and fifth year since the diagnosis. Cancer type was categorized into acute lymphoblastic leukemia and lymphoblastic lymphoma (ALL/LBL), CNS tumors, and all other malignant neoplasms. Treatment duration for ALL/LBL is considerably long, whereas CNS is characterized by both high treatment burden and effects on the CNS.^20^ Child's age at diagnosis was categorized as below 10 years old versus 10–19 years old. We also recorded whether there was at least one underage sibling living in the household, whether the parent was living together with the child's other biological parent, and the parent's education level (tertiary, secondary, and basic), all measured at the year of child's cancer diagnosis. Parent's age and household disposable income (annual quintiles within the total Finnish population) were recorded annually.

After presenting descriptive results, we examined within‐individual changes in parents' psychotropic medication use according to time since child's cancer diagnosis and parent's gender using linear probability models with the individual fixed‐effects estimator. The fixed‐effects estimator is based only on within‐individual variation: we compare each parent's annual probability of psychotropic medication use over time since the child's cancer diagnosis to their own annual probability of psychotropic medication use during the 5‐year period before the child's diagnosis. Instead of comparing parents of children diagnosed with cancer to parents of children not diagnosed with cancer that have been matched on observed characteristics, the fixed‐effects approach uses each parent as his or her own control. Thus, in addition to controlling for all observed differences between parents, the fixed‐effects approach allows us to control for all stable unmeasured characteristics, such as parent's history of mental health and previous psychotropic medication use.^39^ To assess whether changes in parents' psychotropic medication use differed by child's cancer type, child's age at diagnosis, presence of siblings, and parent's living arrangement and education, we modeled the changes separately by each characteristic. All models were controlled for parent's age, as there is an overall increasing trend in psychotropic medication use over time and with age (for individuals, age and calendar time increase in parallel).^44^, ^45^ In additional analyses, we replicated these models controlling for changes in household disposable income.

## 3 RESULTS

With only the parents of children who survived at least a year after their diagnosis included, 90% of the children were still alive 5 years after the diagnosis (Table 1). One‐fifth of children were diagnosed with a CNS cancer and another fifth with ALL/LBL. About half of the children were younger than 10 years old at diagnosis. Psychotropic medication use was more common among mothers than among fathers already before the child's diagnosis, 11.2% versus 7.3%. Mothers of older and only children, mothers not living together with the child's father, and mothers with only basic education had higher psychotropic medication use both 1 year before and 1 year after the diagnosis compared to other mothers. Differences between groups were much smaller among fathers.

**TABLE 1:** Parents' characteristics and psychotropic medication prevalence 1 year before and 1 year after the child's cancer diagnosis

	Mothers (*N* = 3266)			Fathers (*N* = 2687)		
	Distribution at the time of diagnosis	Medication prevalence year before diagnosis, %	Medication prevalence year after diagnosis, %	Distribution at the time of diagnosis	Medication prevalence year before diagnosis, %	Medication prevalence year after diagnosis, %
Parent's mean age, years	39.5	11.2	17.9	41.8	7.3	10.4
Child deceased within 5 years, %						
No	89.8	11.1	17.7	90.3	7.4	10.5
Yes	10.2	11.7	20.1	9.7	6.9	9.6
Child's cancer type^a^, %						
ALL/LBL	21.5	9.8	16.5	22.2	5.5	10.4
CNS	21.8	11.4	17.3	21.5	6.8	11.3
Other	56.7	11.6	18.7	56.3	8.3	10.1
Child's age at diagnosis, %						
0–9 years	52.7	8.6	16.2	54.9	6.6	10.5
10–19 years	47.3	14.0	19.8	45.1	8.2	10.4
Presence of underage sibling, %						
Yes	67.1	10.0	17.0	69.2	7.3	10.8
No	32.9	13.7	19.7	30.8	7.4	9.6
Parent lives with child's other biological parent, %						
Yes	75.5	9.6	16.2	91.7	7.4	10.2
No	24.5	16.0	23.1	8.3	7.2	13.0
Parent's education, %						
Basic	13.2	13.7	22.1	16.0	7.0	8.6
Secondary	40.3	9.5	16.0	45.7	7.6	10.7
Tertiary	46.5	11.9	18.4	38.3	7.2	10.9
Household disposable income quintile, %						
First (lowest)	14.5	11.3	18.2	10.9	9.2	11.0
Second	22.6	12.1	20.2	20.3	7.7	10.1
Third	24.0	10.9	15.0	25.3	6.8	12.5
Fourth	22.4	9.5	16.8	24.8	6.7	9.0
Fifth (highest)	16.5	11.9	20.3	18.7	6.9	9.6

^a^ Child's cancer type was categorized into acute lymphoblastic leukemia and lymphoblastic lymphoma (ALL/LBL), central nervous system tumors (CNS), and all other malignant neoplasms.

**FIGURE 1:**
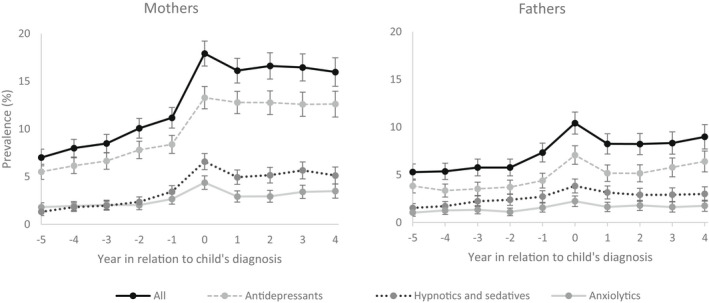
Annual prevalence of parents' psychotropic medication use before and after child's cancer diagnosis by medication type and parent's gender

In the sample, there was a clear increase in the use of psychotropic medication immediately after the child's diagnosis among mothers, followed by a stronger decline in anxiolytics as well as hypnotics and sedatives than antidepressants (Figure 1). Among fathers, the changes in antidepressant use were instead larger than those observed in the use of anxiolytics, hypnotics, and sedatives. Nevertheless, the overall pattern of changes––a clear increase followed by a decline thereafter––was similar irrespective of medication type, and in further within‐individual analyses we thus investigated all psychotropic medications as one group. Already during the 5 years preceding the child's cancer diagnosis, there was an increasing trend in parents' psychotropic medication use, and after the diagnosis psychotropic medication use appeared to remain persistently elevated, particularly among mothers. However, these findings partly reflect the overall increasing trend in psychotropic medication use over time and with age.

In the fixed‐effects models that control for the within‐individual trends in psychotropic medication use with age (and over time) in addition to all stable characteristics between individuals, psychotropic medication use was elevated by 6.0 percentage points immediately after the child's diagnosis among mothers but returned to the pre‐diagnosis level by the end of the 5‐year follow‐up (Table 2). Among fathers, psychotropic medication use was elevated by 3.2 percentage‐points immediately after child's diagnosis and returned to pre‐diagnosis level already during the second year after diagnosis. Among both mothers and fathers, psychotropic medication use increased in the first year since the child's diagnosis irrespective of the cancer type, child's age at diagnosis and other family characteristics. Thereafter, among mothers whose child was diagnosed with a CNS cancer, psychotropic medication use remained persistently elevated, and among fathers whose child was diagnosed with ALL/LBL, the pre‐diagnosis level in psychotropic medication use was reached only at the end of the 5‐year follow‐up period. Among mothers who had other under‐aged children at home or whose diagnosed child was younger than 10 years old, the return to previous levels was also slower compared to other mothers, and the pre‐diagnosis level in medication use was reached only at the end of the 5‐year follow‐up. Mothers in intact two‐parent families also showed a slightly slower decline in medication use than other mothers. Interestingly, psychotropic medication prevalence seemed to return to pre‐diagnosis level somewhat more slowly among mothers with secondary education compared to mothers with either tertiary or only basic education. Among fathers, psychotropic medication use returned to pre‐diagnosis levels after the first year since the child's diagnosis irrespective of the child's age at diagnosis or other family characteristics. Adjusting for household disposable income had negligible effect on any of the associations among both mothers and fathers (Table S2). The results were also similar if we excluded parents of children deceased during the 5‐year follow‐up (Table S3). Furthermore, conclusions remained similar if we used an annual categorization of time since the child's cancer diagnosis (Table S4).

**TABLE 2:** Predicted change^a^ in parents' psychotropic medication use by time since child's diagnosis and family characteristics; age‐adjusted fixed‐effects models

	Mothers			Fathers		
	Time since child's diagnosis			Time since child's diagnosis		
	First year	Second to third year	Fourth to fifth year	First year	Second to third year	Fourth to fifth year
ALL	6.0 (4.8–7.2)	3.0 (1.6–4.4)	1.2 (−0.7 to 3.0)	3.2 (2.1–4.2)	0.4 (−0.8 to 1.6)	0.1 (−1.4 to 1.7)
Cancer type^b^						
ALL/LBL	5.6 (3.1–8.0)	1.6 (−1.2 to 4.5)	−1.5 (−5.4 to 2.4)	5.2 (2.9–7.5)	3.6 (1.0–6.2)	3.0 (−0.3 to 6.4)
CNS	5.1 (2.4–7.8)	4.0 (0.9–7.2)	4.4 (0.2–8.5)	4.4 (1.9–6.9)	0.5 (−2.0 to 3.1)	−0.1 (−3.3 to 3.1)
Other	6.5 (5.0–8.1)	3.1 (1.3–4.9)	1.0 (−1.4 to 3.4)	1.9 (0.6–3.3)	−0.9 (−2.4 to 0.6)	−0.9 (−3.1 to 1.3)
Child's age at diagnosis						
0–9 years	6.8 (5.2–8.4)	4.4 (2.5–6.3)	3.6 (1.1–6.2)	4.2 (2.7–5.6)	1.1 (−0.5 to 2.7)	−0.1 (−2.2 to 2.0)
10–19 years	5.2 (3.4–7.0)	1.4 (−0.6 to 3.4)	−1.5 (−4.2 to 1.2)	2.0 (0.5–3.6)	−0.5 (−2.2 to 1.2)	0.3 (−2.1 to 2.7)
Presence of underage sibling						
Yes	6.6 (5.1–8.0)	3.9 (2.2–5.5)	2.5 (0.3–4.7)	3.5 (2.2–4.8)	0.1 (−1.3 to 1.5)	−0.7 (−2.6 to 1.2)
No	4.9 (2.7–7.1)	1.1 (−1.3 to 3.6)	−1.5 (−5.0 to 1.9)	2.5 (0.6–4.4)	1.1 (−0.8 to 3.1)	1.9 (−0.9 to 4.8)
Parent lives with child's other biological parent						
Yes	5.9 (4.6–7.2)	3.1 (1.7–4.6)	1.9 (−0.1 to 3.9)	3.1 (2.0–4.1)	0.5 (−0.7 to 1.7)	0.2 (−1.4 to 1.8)
No	6.5 (3.7–9.3)	2.5 (−0.8 to 5.7)	−1.0 (−5.3 to 3.3)	4.5 (−0.2 to 9.3)	−0.2 (−5.1 to 4.6)	−0.7 (−7.2 to 5.9)
Parent's education						
Basic	6.9 (3.7–10.2)	2.8 (−1.1 to 6.8)	−0.1 (−5.3 to 5.2)	2.2 (−0.3 to 4.7)	0.3 (−2.5 to 3.0)	0.2 (−3.7 to 4.1)
Secondary	5.5 (3.7–7.3)	3.2 (1.2–5.2)	3.1 (0.3–5.9)	2.8 (1.2–4.4)	−0.2 (−2.0 to 1.7)	−1.0 (−3.3 to 1.4)
Tertiary	6.2 (4.4–8.1)	2.8 (0.8–4.9)	−0.2 (−3.0 to 2.6)	4.1 (2.4–5.7)	1.2 (−0.6 to 3.0)	1.4 (−1.2 to 4.0)

^a^ Difference in prevalence compared to pre‐diagnosis level (annual prevalence during the 5‐year period); percentage points with 95% confidence intervals.

^b^ Child's cancer type was categorized into acute lymphoblastic leukemia and lymphoblastic lymphoma (ALL/LBL), central nervous system tumors (CNS), and all other malignant neoplasms.

## 4 DISCUSSION

Having a child diagnosed with cancer clearly increased the prevalence of parents' psychotropic medication use, from 11% to 18% among mothers, and from 7% to 10% among fathers. The initial shock of having a child diagnosed with cancer thus seems to affect the mental well‐being of mothers more strongly than fathers, at least in absolute terms. The increase in medication use was similar regardless of cancer type, child's age at diagnosis, and other family characteristics with potential impact on caregiving strain. This could be expected, as difficulties in combining the care of the child with other responsibilities are likely to emerge only later on.^14^

The increase in psychotropic medication use corroborates findings from a previous Danish study that showed an increased probability to initiate psychotropic medication use following child's diagnosis.^37^ However, to the best of our knowledge, our study is the first to assess changes in psychotropic medication use over time since the child's diagnosis. We showed that mothers not only experience a larger increase in psychotropic medication use in the first year after their child's diagnosis compared to fathers, but that among mothers medication use also declines more slowly thereafter. The higher prevalence of psychotropic medication use among mothers than fathers already before the child's cancer diagnosis reflects women's higher tendency to use psychotropic medication compared to men.^45^ Although women are more likely than men to suffer from anxiety disorders^46^ and depression,^47^ psychotropic medication use is more common among women than men even after adjustment for mental health status.^48^ This gender bias in psychotropic medication use may thus partly explain the higher increase in psychotropic medication use following child's cancer diagnosis among mothers, suggesting that women are more likely to seek and receive care than fathers.

The only group of fathers whose psychotropic medication use did not return to pre‐diagnosis levels by the second year of follow‐up were those whose child was diagnosed with ALL/LBL. Treatment durations for ALL/LBL are long––from 2 to 2.5 years––and previous studies have suggested that frequent and prolonged hospital care periods have a particularly strong adverse effects on parents' mental health.^10^, ^12^, ^21^, ^22^, ^23^ Interestingly, among mothers of children diagnosed with ALL/LBL the decline in psychotropic medication use following child's diagnosis was somewhat faster, whereas among mothers of children diagnosed with a CNS cancer psychotropic medication use remained persistently elevated during the 5‐year follow‐up. In addition to a high treatment burden, CNS cancers have effects on the CNS which may increase the overall burden of care in the long term. Compared to other mothers, the decline in psychotropic medication use over time since the child's cancer diagnosis was also slower among mothers whose child was young at the time of di

agnosis or who had multiple children. The slower decline and persistent effects suggest that an increased burden of care within the family disproportionately affects the mental well‐being of mothers. Parents of children with cancer often follow traditional gender roles, with mothers taking the main responsibility of care not only for the diagnosed child, but also for the healthy siblings.^14^, ^33^, ^49^ Difficulties in combining parenting responsibilities and work may also be more pronounced among mothers.^14^ In Sweden, while both mothers and fathers take time off from work around the time of their child's diagnosis, fathers return to work earlier while mothers continue to be on leave,^50^, ^51^ which is reflected on the lower long‐term employment status and income of mothers of children with cancer even in the gender‐egalitarian Nordic countries.^27^ However, in our study, changes in household income had no impact on the effect of the child's cancer on mothers' psychotropic medication use, potentially because the Finnish welfare state compensates for the income losses experienced by parents and because household income was not very strongly associated with psychotropic medication use, particularly among women.

Our findings did not support the idea that single‐parents or socioeconomically disadvantaged parents with only basic education are particularly prone to adverse mental health effects following their child's cancer diagnosis, although one should note the high prevalence of psychotropic medication use among single and low‐educated mothers already before the child's diagnosis. Instead, mothers with secondary education were the ones to recover somewhat more slowly than both less and more educated mothers. It could be that mothers with secondary education more often work in environments that are more demanding than jobs that only require basic education, but at the same time the jobs may be less flexible compared to workplaces of tertiary‐educated women. Job demands and flexibility impact the balance between work and family among parents of ill children,^14^, ^30^, ^31^ and may thus at least partly explain the slow recovery among mothers with secondary education. Medication use also declined somewhat more slowly among mothers in intact two‐parent families than among single mothers. However, this could be explained by higher caregiving strain since mothers in two‐parent families more often have younger, and more children at home.

At the population level, the increase in medication use immediately after child's diagnosis was evident for anxiolytics, hypnotics, and sedatives, as well as antidepressants. Clinicians have often used anxiolytics to treat symptoms of anxiety,^41^ although antidepressants are the recommended first line of pharmacological treatment for both depressive and anxiety disorders.^42^, ^43^ Interestingly, in a previous Danish study, only the risk of a first prescription of anxiolytics or hypnotics, but not of antidepressants, was elevated after child's diagnosis.^37^ A potential explanation is that the study excluded all parents who had been prescribed any psychotropic medication during the 3 years before their child's diagnosis. Given the relatively high prevalence of antidepressant use, this exclusion is likely to underestimate the effect of child's cancer diagnosis on parents' antidepressant use. To overcome this limitation, we included all parents and used the fixed‐effects approach to account for their previous history of psychotropic medication use.^39^

### 4.1 Limitations

Our study benefits from longitudinal population‐representative data that enabled us to follow a large cohort of parents. The data are of high quality with no recall bias and selective attrition, which are major limitations in surveys. However, while in Finland both medical care and medication costs are subsidized, making psychotropic medications available to all residents, not all parents with mental health problems seek treatment. Family caregivers of adult cancer patients often underuse mental health services,^52^, ^53^ and the same may be true for parents of children with cancer, although comprehensive monitoring of parents' mental health has been proposed as a standard in the treatment of children with cancer.^54^ It is also possible that parents with milder symptoms are offered other treatments and forms of support such as counseling and peer support instead of psychotropic medications, although patients seeking help for depressive symptoms in primary care are commonly offered antidepressants.^55^, ^56^ Psychotropic medications may also be used for other than psychiatric indications, although less so in working age.^55^ We also have no reason to expect that child's cancer diagnosis would particularly increase parents' psychotropic medication use for nonpsychiatric indications. The observed increase in psychotropic medication use immediately after child's diagnosis is likely an underestimate of the increase in psychological distress. However, among those parents who have initiated psychotropic medication use, we expect further changes in medication use to accurately estimate underlying changes in parents' mental well‐being. Nevertheless, it is possible that the purchased psychotropic medications are not actually used, although we would expect a purchase to reflect a strong intention of use as the reimbursement for medication expenses does not cover the full costs. Finally, although we were able to examine how several family characteristics and child's cancer type moderate changes in parents' psychotropic medication use following their child's diagnosis, we could not measure the course and duration of treatment or later relapses. Previous research has linked relapses with parents' increased probability to initiate the use of hypnotics,^37^ and frequent and prolonged periods of hospital care have been associated with strong adverse effects on parents' mental health.^10^, ^12^, ^21^, ^22^ We thus encourage future studies to assess how child's treatment and potential relapses are associated with changes in parents' psychotropic medication use, taking into account parental characteristics and the family context. Future research could also employ a dyadic approach to examine how changes in one parent's psychotropic medication use are associated with changes in the other parent's psychotropic medication use.

## 5 CONCLUSIONS

Psychotropic medication use is clearly elevated among parents of children with cancer, particularly among mothers who also appear to recover more slowly than fathers. Support to parents should be available over an extended time‐period after the child's diagnosis, and having a child with ALL/LBL or CNS cancer, child's young age at diagnosis, as well as family characteristics such as presence of underage siblings should guide identification of families with a prolonged need of support.

## CONFLICT OF INTEREST

None declared.

## AUTHOR CONTRIBUTIONS

Niina Metsä‐Simola: Conceptualization, Methodology, Formal analysis, Writing – original draft preparation, Writing – review and editing, Supervision, and Project Administration. Hanna Remes: Conceptualization, Writing – review and editing. Elina Hiltunen: Conceptualization, Formal analysis, and Writing – review and editing. Pekka Martikainen: Conceptualization, Writing – review and editing.

## ETHICS STATEMENT

The study was performed under the Ethics Committee of Statistics Finland's permission TK‐53‐1121‐18.

## Supporting information


Figure S1

Table S1
Table S2
Table S3
Table S4

## Data Availability

Researchers may apply for permission to use the data. Permissions from both Statistics Finland (socio‐demographic and family data) and Findata (health data) are needed. After permission has been granted, data will be available in the remote access system provided by the register holders. The code used in the analyses of this paper may then be uploaded to the remote access system by contacting the authors.
