# Supplementary material for: Changes in parents' psychotropic medication use following child's cancer diagnosis: A fixed‐effects register‐study in Finland
Source: Cancer Med. 2022 Mar 28;11(16):3145–55. doi: 10.1002/cam4.4662 (PMC9385598; doi:10.1002/cam4.4662)

Supplementary Figure 1. Sample exclusions, mothers and fathers whose child was diagnosed with cancer.

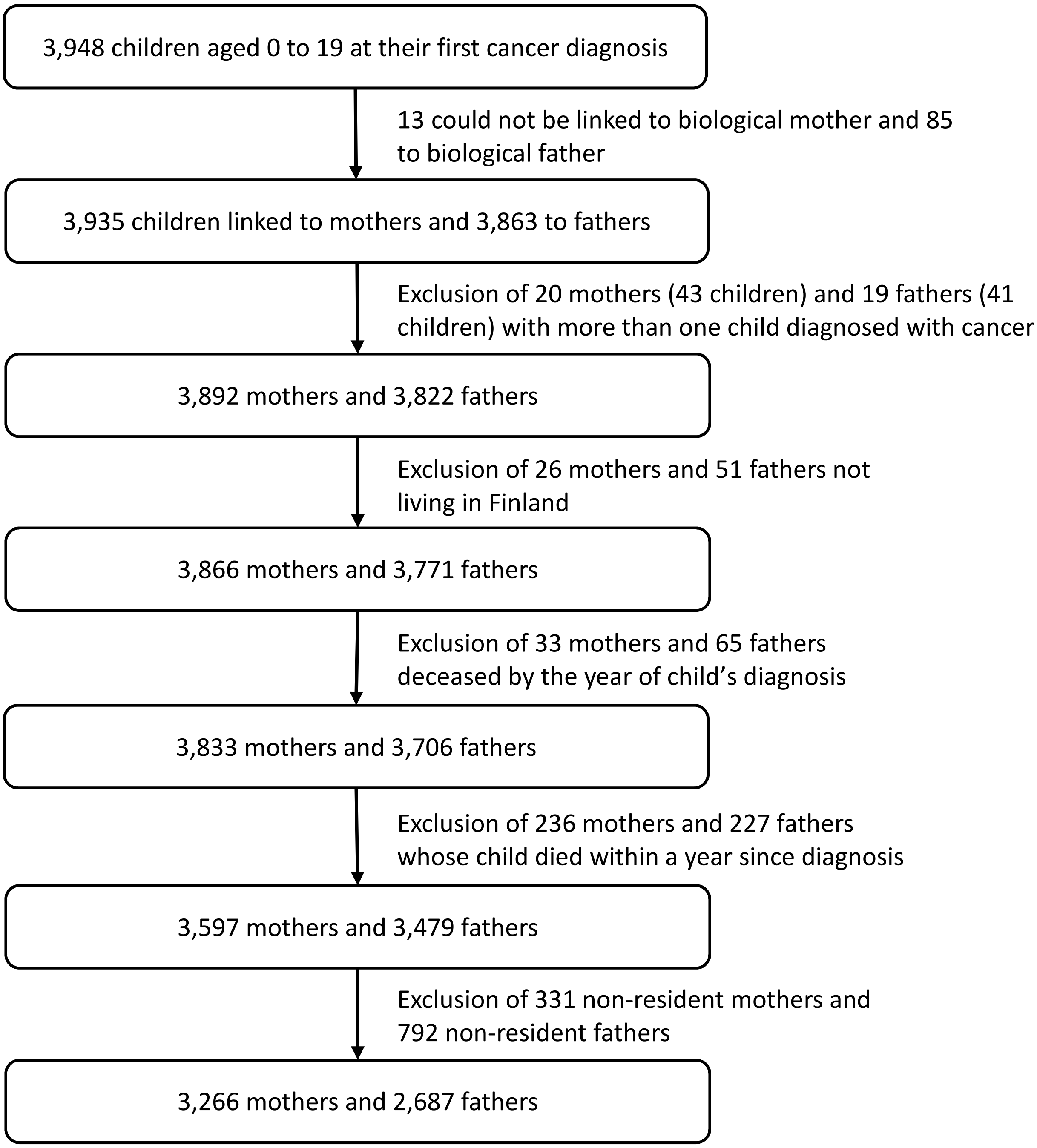

Supplement: Supplementary file 1 — Figure S1 [file CAM4-11-3145-s002.pdf]
