# Supplementary material for: Changes in parents' psychotropic medication use following child's cancer diagnosis: A fixed‐effects register‐study in Finland
Source: Cancer Med. 2022 Mar 28;11(16):3145–55. doi: 10.1002/cam4.4662 (PMC9385598; doi:10.1002/cam4.4662)
Supplement: Supplementary file 2 — Table S1 Table S2 Table S3 Table S4 [file CAM4-11-3145-s001.docx]

| Supplementary Table 1. Predicted change^†^ in parents' psychotropic medication use by time since child's diagnosis and parent's living arrangement; age-adjusted fixed-effects models | | | | | | | | | |
| --- | --- | --- | --- | --- | --- | --- | --- | --- | --- |
|  | |  |  |  |  |  |  |  |  |
|  |  |  |  |  |  |  |  |  |  |
|  |  | TIME SINCE DIAGNOSIS (YEARS) | | | | | | | |
|  |  |  |  |  |  |  |  |  |  |
|  |  | First year | |  | Second to third year | |  | Fourth to fifth year | |
| MOTHERS | |  |  |  |  |  |  |  |  |
|  | Lives with child and child's other biological parent | 5.8 | (4.6-7.2) |  | 3.1 | (1.7-4.6) |  | 1.9 | (-0.1-3.9) |
|  | Lives with child- but not with the other biological parents | 6.5 | (3.7-9.3) |  | 2.5 | (-0.8-5.7) |  | -1.0 | (-5.3-3.3) |
|  | Does not live with child | 3.5 | (-0.3-7.2) |  | 3.0 | (-1.6-7.7) |  | 4.3 | (-2.0-10.6) |
| FATHERS | |  |  |  |  |  |  |  |  |
|  | Lives with child and child's other biological parent | 3.1 | (2.0-4.1) |  | 0.5 | (-0.7-1.7) |  | 0.2 | (-1.4-1.8) |
|  | Lives with child- but not with the other biological parents | 4.5 | (-0.2-9.3) |  | -0.2 | (-5.1-4.6) |  | -0.7 | (-7.2-5.9) |
|  | Does not live with child | 1.1 | (-1.0-3.2) |  | 0.3 | (-2.3-2.9) |  | -0.3 | (-4.0-3.4) |
|  |  |  |  |  |  |  |  |  |  |
| ^†^Difference in prevalence compared to pre-diagnosis level; percentage points with 95% confidence intervals | | | | | | | | |  |

Supplementary Table 2. Predicted change^†^ in parents' psychotropic medication use by time since child's diagnosis and family characteristics; fixed-effects models adjusted for age and income

|  |  | MOTHERS | | | | | |  | FATHERS | | | | | |  |
| --- | --- | --- | --- | --- | --- | --- | --- | --- | --- | --- | --- | --- | --- | --- | --- |
|  |  |  |  |  |  |  |  |  |  |  |  |  |  |  | |
|  |  |  | TIME SINCE DIAGNOSIS (YEARS) | | | | |  | TIME SINCE DIAGNOSIS (YEARS) | | | | | |  |
|  |  |  |  |  |  |  |  |  |  |  |  |  |  |  | |
|  | | First year | | Second to  third year | | Fourth to  fifth year | |  | First year | | Second to  third year | | Fourth to  fifth year | | |
|  |  |  |  |  |  |  |  |  |  |  |  |  |  |  | |
| ALL |  | 6.0 | (4.8-7.2) | 2.9 | (1.5-4.3) | 1.1 | (-0.7-3.0) |  | 3.2 | (2.1-4.2) | 0.4 | (-0.7-1.6) | 0.2 | (-1.4-1.8) | |
| Cancer type^‡^ | |  |  |  |  |  |  |  |  |  |  |  |  |  | |
|  | ALL/LBL | 5.5 | (3.1-8.0) | 1.5 | (-1.5-4.4) | -1.6 | (-5.6-2.4) |  | 5.3 | (2.9-7.6) | 3.6 | (1.1-6.2) | 3.1 | (-0.3-6.5) | |
|  | CNS | 4.9 | (2.2-7.6) | 3.9 | (0.8-7.1) | 4.4 | (0.1-8.6) |  | 4.3 | (1.8-6.7) | 0.5 | (2.1-3.0) | -0.1 | (-3.3-3.0) | |
|  | Other | 6.6 | (5.0-8.1) | 3.1 | (1.3-4.9) | 1.0 | (-1.5-3.4) |  | 1.9 | (0.6-3.3) | -0.8 | (-2.4-0.7) | -0.8 | (-3.0-1.4) | |
| Child's age at diagnosis | |  |  |  |  |  |  |  |  |  |  |  |  |  | |
|  | 0 to 9 years | 6.7 | (5.1-8.4) | 4.3 | (2.4-6.2) | 3.4 | (0.9-6.0) |  | 4.2 | (2.7-5.6) | 1.1 | (-0.4-2.8) | 0.1 | (-2.0-2.2) | |
|  | 10 to 19 years | 5.2 | (3.4-6.9) | 1.4 | (-0.6-3.4) | -1.5 | (-4.2-1.3) |  | 2.0 | (0.5-3.5) | -0.5 | (-2.2-1.2) | 0.3 | (-2.0-2.8) | |
| Presence of underage sibling | |  |  |  |  |  |  |  |  |  |  |  |  |  | |
|  | Yes | 6.5 | (5.1-7.9) | 3.8 | (2.1-5.4) | 2.4 | (0.2-4.6) |  | 3.5 | (2.2-4.8) | 0.1 | (-1.3-1.6) | -0.5 | (-2.4-1.4) | |
|  | No | 4.9 | (2.8-7.1) | 1.2 | (-1.2-3.7) | -1.3 | (-4.8-2.2) |  | 2.4 | (0.6-4.3) | 1.0 | (-1.0-3.0) | 1.8 | (-1.0-4.7) | |
| Parent lives with child's other biological parent | |  |  |  |  |  |  |  |  |  |  |  |  |  | |
|  | Yes | 5.9 | (4.6-7.2) | 3.1 | (1.6- 4.6) | 1.8 | (-0.2- 3.9) |  | 3.0 | (2.0- 4.1) | 0.5 | (-0.7- 1.7) | 0.3 | (-1.4-1.9) | |
|  | No | 6.3 | (3.5-9.1) | 2.2 | (-1.1- 5.5) | -1.3 | (-5.8- 3.1) |  | 4.7 | (-0.1- 9.5) | 0.2 | (-4.8- 5.0) | -0.1 | (-6.7-6.5) | |
| Parent's education | |  |  |  |  |  |  |  |  |  |  |  |  |  | |
|  | Basic | 6.4 | (3.1-9.8) | 2.1 | (-2.0-6.2) | -0.6 | (-6.1-4.8) |  | 2.2 | (-0.1-4.5) | 0.7 | (-1.9-3..4) | 0.1 | (-2.7-4.9) | |
|  | Secondary | 5.5 | (3.7-7.3) | 3.2 | (1.2-5.3) | 3.1 | (0.2-5.9) |  | 2.7 | (1.1-4.4) | -0.3 | (-2.1-1.5) | -1.1 | (-3.5-1.2) | |
|  | Tertiary | 6.2 | (4.4-8.1) | 2.8 | (0.7-4.9) | -0.2 | (-3.0-2.6) |  | 4.1 | (2.5-5.8) | 1.2 | (-0.7-3.0) | 1.4 | (-1.2-4.0) | |

^†^Difference in prevalence compared to pre-diagnosis level; percentage points with 95% confidence intervals

^‡^Child’s cancer type was categorized into acute lymphoblastic leukemia and lymphoblastic lymphoma (ALL/LBL), central nervous system tumors (CNS) and all other malignant neoplasms

Supplementary Table 3. Predicted change^†^ in parents' psychotropic medication use by time since child's diagnosis and family characteristics; age-adjusted fixed-effects models with parents of deceased children excluded

|  |  | MOTHERS | | | | | |  | FATHERS | | | | | | | | | |
| --- | --- | --- | --- | --- | --- | --- | --- | --- | --- | --- | --- | --- | --- | --- | --- | --- | --- | --- |
|  |  |  |  |  |  |  |  |  |  | |  | |  |  | |  |  | |
|  |  | TIME SINCE DIAGNOSIS (YEARS) | | | | | |  | TIME SINCE DIAGNOSIS (YEARS) | | | | | | | | | |
|  |  |  |  |  |  |  |  |  |  | |  | |  |  | |  |  | |
|  | | First year | | Second to  third year | | Fourth to  fifth year | |  | | First year | | Second to  third year | | | Fourth to  fifth year | | |  |
|  |  |  |  |  |  |  |  |  |  | |  | |  |  | |  |  | |
| ALL |  | 5.8 | (4.6-7.1) | 2.8 | (1.4-4.3) | 1.3 | (-0.6-3.2) |  | 3.3 | | (2.2-4.4) | | 0.3 | (-0.9-1.5) | | 0.2 | (-1.4-1.8) | |
| Cancer type^‡^ | |  |  |  |  |  |  |  |  | |  | |  |  | |  |  | |
|  | ALL/LBL | 6.1 | (3.5-8.7) | 1.8 | (-1.2-4.8) | -1.3 | (-5.4-2.7) |  | 5.1 | | (2.6-7.6) | | 3.3 | (0.7-5.9) | | 2.6 | (-0.8-6.0) | |
|  | CNS | 4.3 | (1.5-7.2) | 4.6 | (1.2-7.9) | 5.3 | (0.9-9.6) |  | 4.7 | | (2.0-7.3) | | 0.9 | (-1.7-3.6) | | 0.5 | (-2.9-3.8) | |
|  | Other | 6.3 | (4.6-7.9) | 2.6 | (0.8-4.4) | 0.8 | (-1.7-3.3) |  | 2.0 | | (0.6-3.4) | | -1.1 | (-2.6-0.4) | | -0.8 | (-3.1-1.4) | |
| Child's age | |  |  |  |  |  |  |  |  | |  | |  |  | |  |  | |
|  | 0 to 9 years | 6.7 | (5.1-8.4) | 4.5 | (2.6-6.5) | 3.9 | (1.3-6.5) |  | 4.0 | | (2.5-5.6) | | 1.0 | (-0.6-2.6) | | 0.0 | (-2.2-2.1) | |
|  | 10 to 19 years | 4.8 | (2.9-6.7) | 1.0 | (-1.1-3.0) | -1.6 | (-4.4-1.2) |  | 2.3 | | (0.7-3.9) | | -0.6 | (-2.3-1.1) | | 0.5 | (-2.0-2.9) | |
| Presence of underage sibling | |  |  |  |  |  |  |  |  | |  | |  |  | |  |  | |
|  | Yes | 6.5 | (5.0-8.0) | 3.8 | (2.1-5.6) | 2.5 | (0.2-4.8) |  | 3.7 | | (2.4-5.0) | | 0.2 | (-1.3-1.7) | | -0.4 | (-2.4-1.6) | |
|  | No | 4.4 | (2.2-6.6) | 0.9 | (-1.6-3.4) | -1.1 | (-4.6-2.5) |  | 2.3 | | (0.3-3.2) | | 0.5 | (-1.5-2.5) | | 1.5 | (-1.4-4.4) | |
| Parent lives with child's other biological parent | |  |  |  |  |  |  |  |  | |  | |  |  | |  |  | |
|  | Yes | 5.8 | (4.4-7.1) | 3.2 | (1.6-4.7) | 2.1 | (0.1-4.2) |  | 3.2 | | (2.0-4.3) | | 0.4 | (-0.9-1.6) | | 0.3 | (-1.3-2.0) | |
|  | No | 5.9 | (2.9-8.9) | 1.8 | (-1.6-5.3) | -1.3 | (-5.9-3.4) |  | 4.5 | | (-0.5-9.5) | | -0.4 | (-5.5-4.6) | | -1.4 | (-8.2-5.4) | |
| Parent's education | |  |  |  |  |  |  |  |  | |  | |  |  | |  |  | |
|  | Basic | 6.1 | (2.8-9.4) | 2.1 | (-2.0-6.2) | -0.4 | (-5.8-5.0) |  | 3.0 | | (3.1-5.6) | | 1.1 | (-1.7-3.8) | | 1.2 | (-2.7-5.2) | |
|  | Secondary | 5.5 | (3.6-7.4) | 3.1 | (0.9-5.2) | 3.1 | (0.2-6.0) |  | 2.7 | | (1.0-4.4) | | -0.6 | (-2.4-1.3) | | -1.1 | (-3.5-1.3) | |
|  | Tertiary | 6.1 | (4.2-8.0) | 2.9 | (0.7-5.0) | 0.3 | (-2.6-3.1) |  | 4.1 | | (2.4-5.8) | | 1.0 | (-0.9-2.8) | | 1.4 | (-1.3-4.0) | |

^†^Difference in prevalence compared to pre-diagnosis level; percentage points with 95% confidence intervals

^‡^Child’s cancer type was categorized into acute lymphoblastic leukemia and lymphoblastic lymphoma (ALL/LBL), central nervous system tumors (CNS) and all other malignant neoplasms

Supplementary Table 4. Predicted change^†^ in parents' psychotropic medication use by time since child's diagnosis and family characteristics; annual categorization of time since child’s diagnosis, age-adjusted fixed-effects models

|  |  | TIME SINCE DIAGNOSIS (YEARS) | | | | | | | | | | | | | |
| --- | --- | --- | --- | --- | --- | --- | --- | --- | --- | --- | --- | --- | --- | --- | --- |
|  |  |  |  |  |  |  |  |  |  |  |  |  |  |  |  |
|  |  | First year | |  | Second year | |  | Third year | |  | Fourth year | |  | Fifth year | |
| MOTHERS | |  |  |  |  |  |  |  |  |  |  |  |  |  |  |
|  |  |  |  |  |  |  |  |  |  |  |  |  |  |  |  |
| ALL |  | 5.8 | (4.6-7.0) |  | 3.0 | (1.6-4.3) |  | 2.4 | (0.8-4.1) |  | 1.3 | (-0.6-3.3) |  | 0.2 | (-2.0-2.4) |
| Cancer type^‡^ | |  |  |  |  |  |  |  |  |  |  |  |  |  |  |
|  | ALL/LBL | 5.2 | (2.7-7.6) |  | 1.1 | (-1.8-4.0) |  | 0.8 | (-2.9-4.5) |  | -0.6 | (-4.8-3.5) |  | -4.4 | (-9.1-0.2) |
|  | CNS | 4.9 | (2.2-7.6) |  | 4.7 | (1.5-7.8) |  | 2.9 | (-0.8-6.6) |  | 3.7 | (-0.7-8.1) |  | 4.4 | (-0.3-9.1) |
|  | Other | 6.5 | (4.9-8.0) |  | 3.0 | (1.2-4.8) |  | 2.9 | (0.7-5.1) |  | 1.1 | (-1.4-3.7) |  | 0.4 | (-2.4-3.3) |
| Child's age at diagnosis | |  |  |  |  |  |  |  |  |  |  |  |  |  |  |
|  | 0 to 9 years | 6.7 | (5.1-8.3) |  | 4.3 | (2.4-6.2) |  | 4.2 | (1.9-6.5) |  | 3.8 | (1.1-6.4) |  | 3.0 | (0.0-6.0) |
|  | 10 to 19 years | 4.9 | (3.1-6.7) |  | 1.4 | (-0.6-3.4) |  | 0.5 | (-1.9-2.9) |  | -1.4 | (-4.3-1.5) |  | -2.9 | (-6.1-0.2) |
| Presence of underage sibling | |  |  |  |  |  |  |  |  |  |  |  |  |  |  |
|  | Yes | 6.5 | (5.1-7.9) |  | 3.7 | (2.0-5.4) |  | 3.9 | (1.8-6.0) |  | 2.8 | (0.5-5.1) |  | 1.9 | (-0.7-4.5) |
|  | No | 4.5 | (2.3-6.7) |  | 1.4 | (-1.1-3.9) |  | -0.5 | (-3.4-2.4) |  | -1.7 | (-5.3-1.9) |  | -3.2 | (-7.2-0.7) |
| Parent lives with child's other biological parent | | |  |  |  |  |  |  |  |  |  |  |  |  |  |
|  | Yes | 5.8 | (4.5-7.1) |  | 3.0 | (1.5-4.5) |  | 3.0 | (1.2-4.8) |  | 2.1 | (0.0-4.3) |  | 1.0 | (-1.3-3.3) |
|  | No | 6.1 | (3.3-8.9) |  | 2.9 | (-0.3-6.2) |  | 0.9 | (-3.2-5.0) |  | -1.3 | (-5.9, 3.3) |  | -2.3 | (-7.4-2.7) |
| Parent's education | |  |  |  |  |  |  |  |  |  |  |  |  |  |  |
|  | Basic | 6.7 | (3.4-9.9) |  | 3.0 | (-1.1-7.1) |  | 1.7 | (-2.9-6.4) |  | -0.2 | (-5.6-5.2) |  | -1.2 | (-7.4-5.1) |
|  | Secondary | 5.4 | (3.6-7.2) |  | 3.2 | (1.2-5.2) |  | 3.0 | (0.5-5.5) |  | 3.2 | (0.2-6.1) |  | 2.8 | (-0.5-6.0) |
|  | Tertiary | 6.0 | (4.2-7.8) |  | 2.7 | (0.6-4.8) |  | 2.2 | (-0.4-4.8) |  | 0.1 | (-2.9-3.0) |  | -1.6 | (-4.9-1.6) |
|  |  |  |  |  |  |  |  |  |  |  |  |  |  |  |  |
| FATHERS | |  | |  |  | |  |  | |  |  | |  |  | |
|  |  |  |  |  |  |  |  |  |  |  |  |  |  |  |  |
| ALL |  | 3.2 | (2.1-4.2) |  | 0.6 | (-0.6-1.8) |  | 0.1 | (-1.3-1.6) |  | -0.1 | (-1.7-1.6) |  | 0.3 | (-1.6-2.1) |
| Cancer type^‡^ | |  |  |  |  |  |  |  |  |  |  |  |  |  |  |
|  | ALL/LBL | 5.1 | (2.8-7.4) |  | 3.9 | (1.1-6.6) |  | 3.0 | (0.0-6.0) |  | 2.8 | (-0.7-6.3) |  | 2.7 | (-1.1-6.5) |
|  | CNS | 4.3 | (1.8-6.8) |  | 0.6 | (-2.0-3.3) |  | 0.3 | (-3.0-3.5) |  | -0.2 | (-3.5, 3.1) |  | -0.3 | (-4.3-3.8) |
|  | Other | 1.9 | (0.6-3.3) |  | -0.7 | (-2.2-0.8) |  | -1.0 | (-2.9-0.9) |  | -1.1 | (-3.4-1.1) |  | -0.5 | (-3.1-2.0) |
| Child's age at diagnosis | |  |  |  |  |  |  |  |  |  |  |  |  |  |  |
|  | 0 to 9 years | 4.1 | (2.6-5.6) |  | 1.3 | (-0.4-3.0) |  | 0.8 | (-1.1-2.7) |  | -0.2 | (-2.4-2.0) |  | -0.1 | (-2.5-2.3) |
|  | 10 to 19 years | 2.0 | (0.5-3.6) |  | -0.3 | (-2.0-1.4) |  | -0.7 | (-2.8-1.5) |  | 0.1 | (-2.4-2.6) |  | 0.7 | (-2.3-3.6) |
| Presence of underage sibling | |  |  |  |  |  |  |  |  |  |  |  |  |  |  |
|  | Yes | 3.5 | (2.2-4.8) |  | 0.2 | (-1.3-1.7) |  | 0.0 | (-1.8-1.7) |  | -0.8 | (-2.8-1.2) |  | -0.6 | (-2.8-1.7) |
|  | No | 2.4 | (0.6-4.3) |  | 1.5 | (-0.5-3.5) |  | 0.5 | (-2.0-3.0) |  | 1.5 | (-1.3-4.5) |  | 2.1 | (-1.4-5.5) |
| Parent lives with child's other biological parent | | |  |  |  |  |  |  |  |  |  |  |  |  |  |
|  | Yes | 3.1 | (2.0-4.1) |  | 0.7 | (-0.5-1.9) |  | 0.2 | (-1.2-1.7) |  | 0.0 | (-1.7-1.6) |  | 0.5 | (-1.4-2.4) |
|  | No | 4.3 | (-0.5-9.0) |  | -0.4 | (-5.3-4.4) |  | -0.7 | (-6.6-5.1) |  | -0.2 | (-7.0-6.6) |  | -2.4 | (-9.4-4.7) |
| Parent's education | |  |  |  |  |  |  |  |  |  |  |  |  |  |  |
|  | Basic | 2.0 | (-0.5-4.5) |  | 0.3 | (-2.5-3.1) |  | -0.4 | (-3.9-3.2) |  | 0.3 | (-3.8-4.5) |  | -0.8 | (-5.3-3.8) |
|  | Secondary | 2.8 | (1.1-4.4) |  | -0.1 | (-1.9-1.8) |  | -0.3 | (-2.5-1.9) |  | -1.1 | (-3.5-1.4) |  | -0.9 | (-3.6-1.9) |
|  | Tertiary | 4.1 | (2.4-5.8) |  | 1.5 | (-0.4-3.3) |  | 0.9 | (-1.3-3.1) |  | 1.0 | (-1.6-3.6) |  | 2.0 | (-1.0-5.1) |

^†^Difference in prevalence compared to pre-diagnosis level; percentage points with 95% confidence intervals

^‡^Child’s cancer type was categorized into acute lymphoblastic leukemia and lymphoblastic lymphoma (ALL/LBL), central nervous system tumors (CNS) and all other malignant neoplasms
